# Real-world effectiveness of palbociclib in HR+/HER2- metastatic breast cancer: a literature review

**DOI:** 10.2144/fsoa-2023-0074

**Published:** 2024-05-15

**Authors:** Emilie Adrian Christiansen, Iben Kümler

**Affiliations:** 1 Faculty of Health & Medical Sciences, University of Copenhagen, Blegdamsvej 3B, 2200 Copenhagen, Denmark; 2 Department of Oncology, Herlev & Gentofte Hospital, Copenhagen University Hospital, Borgmester Ib Juuls vej 7, 2730 Herlev, Denmark

**Keywords:** CDK4/6 inhibitor, HR+/HER2-, metastatic breast cancer, overall survival, palbociclib, progression-free survival, real-world evidence

## Abstract

Approximately 70% of newly diagnosed breast cancers are of the HR+/HER2- subtype. For the treatment of patients with HR+/HER2- metastatic breast cancer, current guidelines recommend the use of a CDK4/6 inhibitor (palbociclib, ribociclib or abemaciclib) in combination with endocrine therapy. In this review we assess existing literature concerning real-world effectiveness of palbociclib. Survival outcomes in terms of progression-free survival and overall survival are discussed and compared among the included real-world studies and in relation to the phase III PALOMA trials.

## Plain language summary

About 70% of newly diagnosed breast cancers belong to a specific subgroup called hormone receptor positive (HR+)/Human epidermal growth factor receptor 2 negative (HER2-). In cases with metastatic disease, doctors recommend a treatment approach combining drugs such as palbociclib along with hormonal therapy. Our review evaluates how palbociclib performs in patients in real-world practice situations, beyond clinical trial settings. We looked at two key measures: how long the cancer stays controlled (progression-free survival) and overall survival. The results from these real-world studies are discussed and compared to findings in clinical trials.

## Abstract (plain language summary)

Current guidelines recommend the use of a CDK 4/6 inhibitor (CDK4/6i) combined with endocrine therapy (ET) in the treatment of HR+/HER2- metastatic breast cancer (mBC).

The randomized phase III PALOMA trials have reported a significant improvement in progression free survival (PFS) in patients receiving palbociclib in combination with ET in both first- and second-line setting. No significant OS-benefit has been reported.

In recent years, real-world studies have received increased attention as they offer valuable insight on how a given treatment regimen performs in real-life patient populations.

All comparative studies investigating palbociclib in the first-line setting reported a significantly improved PFS outcome in patients receiving palbociclib + AI, thus aligning with findings in PALOMA-2.

Three comparative studies found a significant increase in OS in patients receiving palbociclib + AI in the first-line setting, which contrasts with OS results from the phase III PALOMA-2 trial in which no significant benefit was reported.

PFS outcomes in the real-world studies investigating second-line treatment with palbociclib were similar to the results in PALOMA-3.

A comparative study found a significant OS benefit in patients receiving palbociclib + FVT in second-line, contrary to results from the phase III PALOMA-3 trial, in which no significant benefit was found.

Overall, real-world studies are reporting improved PFS and OS outcomes in patients with HR+/HER2- mBC treated with palbociclib in combination with ET in both first- and second-line setting.

Data from real-world studies on palbociclib showing a significant improvement in OS seem to contradict results from the phase III trials in which no significant OS-benefit was reported.

Real-world results support palbociclib as an effective treatment option in real-world clinical practice. However, additional real-world studies on palbociclib with longer follow-up are still needed.

Given the lack of real-world studies on ribociclib and abemaciclib, it is crucial that studies on these CDK4/6i's are conducted and reported in the future to ensure available real-world data on all three CDK4/6i's.

As the real-world experience with ribociclib and abemaciclib CDK4/6i's increases, it will provide crucial insight on how these CDK4/6i's perform in real-world populations, and whether these results align with findings from the pivotal trials.

Female breast cancer (BC) is now the most commonly diagnosed cancer worldwide with an incidence of 2.3 million new cases in 2020 [1]. Approximately 70% of newly diagnosed breast cancers are of the hormone receptor positive (HR+)/human epidermal receptor 2 (HER2-) subtype [2]. Whereas the 5-year survival rate for primary (non-metastatic) BC is 90.6%, the 5-year survival for metastatic breast cancer (mBC) is only 30% [3].

Endocrine therapy remains the mainstay treatment of HR+/HER2- mBC. However, endocrine resistance plays a major role in the loss of treatment effectiveness. Dysregulation of the cycline dependent kinase 4/6 (CDK4/6) pathway has shown to be one of the mechanisms, from which endocrine resistance can emerge. In the process of overcoming this challenge, CDK4/6 inhibitors (CDK4/6i's) have shown promising results [4]. The CDK4/6i's inhibit the kinase activity of CDK4/6, thereby interrupting the uncontrolled proliferation of cancer cells [5].

Current ESMO guidelines recommend the use of a CDK4/6i in combination with endocrine therapy as first-line therapy in the treatment of HR+/HER2- metastatic breast cancer [6]. Three CDK4/6 inhibitors are currently approved: abemaciclib, palbociclib and ribociclib. palbociclib is approved in combination with an AI as first-line therapy for the treatment of HR+/HER2- metastatic or advanced BC in *postmenopausal* women. The approval was based on results from the randomized phase II PALOMA-1 trial [7] and the double-blinded randomised phase III PALOMA-2 trial [8]. Furthermore, based on results from PALOMA-3 [9], palbociclib is approved in combination with fulvestrant in the treatment of HR+/HER2- advanced or metastatic breast cancer in women having previously progressed on endocrine therapy [10]. All PALOMA trials reported promising results as median progression-free survival (PFS) was significantly improved in patients receiving palbociclib compared with the control group [^7-9^,^11^]. However, PALOMA failed to demonstrate a significant increase in median overall survival (OS) in the palbociclib group, although it was numerically prolonged [^12-15^].

As randomized controlled trials (RCTs) are performed under controlled conditions in highly selective patient populations due to narrow inclusion and exclusion criteria, the generalizability of the results to real-world patient populations may be affected. In recent years, real-world data have received increased attention from healthcare decision makers, as it provides valuable information on how a treatment regimen performs in patients in real-life clinical practice. Real-world studies have the potential to support and validate outcomes from the RCTs, or identify potential gaps between them.

However, the real-world studies do have limitations, particularly related to their observational study design, resulting in increased risk of confounding bias [^16-18^].

To our knowledge, only one other review addressing efficacy of CDK4/6i's in the real-world setting has been published so far [19]. Since then, additional data on real-world effectiveness of the CDK4/6i's have become available. In the present review, we aim to provide an updated overview of the real-world effectiveness of palbociclib in the treatment of HR+/HER2- metastatic breast cancer.

## Methods

A search was conducted in the PubMed database to identify relevant studies. The search was set up by using the block search method with relevant medical subject headings (MeSH) terms and free text words (search strategy is shown in Table 1). Searches were performed up till October 25th, 2022.

**Table 1.** Search strategy.

	BLOCK 1	BLOCK 2	BLOCK 3	BLOCK 4	BLOCK 5
Subject/keyword	Breast cancer	ER-positive	Metastasis	CDK 4/6 inhibitors	Real-world
MeSH	“Breast neoplasm” [MeSH]	“Receptors, Estrogen” [MeSH]	“Neoplasm metastasis” [MeSH]	“Cyclin-dependent kinase inhibitor proteins” [MeSH] OR “Palbociclib” [Supplementary concept] OR “Abemaciclib” [Supplementary concept] OR “Ribociclib” [Supplementary concept]	
	*Search #1*	*Search #4*	*Search #7*	*Search #10*	
Free text	“Breast neoplasm*” OR “Breast cancer” OR “Mamma cancer” OR “Cancer in breast*” OR “Cancer of breast*” OR “Cancer mamma*” OR “Mammary cancer*” OR “Mammary neoplasm*” OR “Breast tumor*” OR “Breast malignant tumor*”	”ER positive” OR “ER-positive” OR “ER+” OR “HR positive” OR “HR-positive” OR “HR+” OR “Estrogen receptor positive” OR “Hormone receptor positive” OR “HR+/HER2-” OR “ER+/HER2-” OR “Oestrogen receptor positive”	“Advanced” OR “Metastasis” OR “Metastase*” OR “Metastatic”	“CDK 4/6 inhibit*” OR “Cyclin-dependent kinase 4/6 inhibit*” OR “Inhibitor* of cyclin-dependent kinase 4/6” OR “Palbociclib” OR “Ibrance” OR “Ribociclib” OR “Kisqali” OR “Abemaciclib” OR “Verzenio”	“Real-world” OR “RWD” OR “RWE” OR “Real-life data” OR “Clinical outcome*” OR “Real-world setting*” OR “Real-world stud*” OR “Real-world clinical practice” OR “Electronic health records”
	*Search #2*	*Search #5*	*Search #8*	*Search #11*	*Search #13*
	*Search* #3 = #1 OR #2	*Search* #6 = #4 OR #5	*Search* #9 = #7 OR #8	*Search* #12 = #10 OR #11	*Search* #13

Final search = #3 AND #6 AND #9 AND #12 AND #13.

The search identified 145 articles, all of which were screened by the authors. The following inclusion criteria were applied: patients with HR+/HER2- mBC; real-world studies; CDK4/6i in combination with AI or FVT, respectively; outcomes reported in terms of PFS and/or OS; studies including >240 patients.

Studies in which it was not possible to distinguish between the line of treatment or the used CDK4/6i in the results were excluded. Studies regarding subsequent therapy after progression on a CDK4/6i were excluded as well.

**Figure 1.**
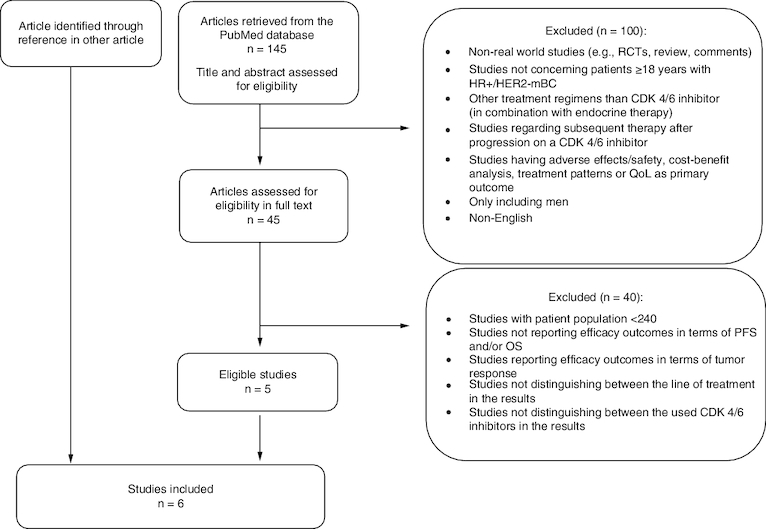
Flow chart of the study selection process.

One study was included through reference in another article (flowchart of the study selection process is shown in Figure 1).

Originally, the intent of this review was to investigate the real-world effectiveness of all three available CDK 4/6 inhibitors. However, the number of real-world studies regarding patients receiving ribociclib or abemaciclib turned out to be limited. The few available studies were either focusing on safety, only included a small population of patients or included more than one CDK 4/6i and failed to distinguish between these in the results. Thus, no real-world studies regarding the efficacy of ribociclib or abemaciclib met the eligibility criteria, resulting in this review only including real-world studies concerning effectiveness of palbociclib.

## Results

The six articles (table of comparison in Tables 2 & 3) included in the present review all address real-world effectiveness of palbociclib in combination with endocrine therapy (ET) in the treatment of HR+/HER2- metastatic breast cancer.

**Table 2.** Comparative studies.

Study (year)	Line of treatment	Treatment regimen	Patients (n)			Follow-up (months)	Median PFS (months [95% CI])			Median OS (months [95% CI])			Ref.
			Unadjusted	sIPTW	PSM	sIPTW	Unadjusted	sIPTW	PSM	Unadjusted	sIPTW	PSM	
Rugo *et al.* (2022)	1st line	PAL + AI vs AI	1324/1564	1572/1137	939/939	23.9/24.5	19.8 (17.9–21.7)/13.9 (12.7–15.2)	**19.3** (17.5–20.7)/**13.9** (12.5–15.2) HR: 0.70 (0.62–0.78), p < 0.0001	19.8 (17.3–21.9)/14.9 (12.9–16.9)	53.4 (48.7–58.6)/40.4 (36.3–44.9)	**49.1** (45.2–57.7)/**43.2** (37.6–48.0) HR: 0.76 (0.65–0.87), p < 0.0001	57.8 (47.2–NE)/43.5 37.6–48.9)	[20]
DeMichele *et al.* (2021)	1st line	PAL + LET vs LET	772/658	839/698	464/464	24.2/23.3	19.7 (17.3–21.9)/11.9 (10.5–13.5)	**20.0** (17.5–21.9)/**11.9** (10.5–13.7) HR: 0.58 (0.49–0.69), p < 0.0001	20.2 (18.2–23.7)/11.9 (10.4–14.5)	NR (NE–NE)/40.3 (34.2–NE)	**NR** (NE–NE)/**43.1** (34.3–NE) HR: 0.66 (0.53–0.82), p = 0.0002	NR (NE–NE)/43.1 (34.2–NE)	[21]
Brufsky *et al.* (2022)	1st line	PAL + LET vs LET (patients with lung and/or liver metastasis)	330/221	321/269	194/194	22.6/22.1	15.4 (12.5–19.5)/10.2 (8.0–11.7)	**16.1** (13.0–20.2)/**9.6** (7.2–11.0) HR: 0.56 (0.45–0.69), p < 0.001	15.7 (12.7–20.2)/9.5 (6.7–10.8)	NR (38.3–NR)/29.4 (25.8–40.8)	**NR** (38.3–NR)/**32.4** (26.0–40.8) HR: 0.58 (0.43–0.77), p < 0.001	NR (42.7–NR)/29.1 (25.3–40.8)	[22]
Ha *et al.* (2022)	1st line	PAL + AI vs AI	778/2452		708/708	PSM: 30.0/119			**17.4** (15.9–20.0)/**11.1** (9.9–13.6) HR: 0.71 (0.64–0.8), p < 0.0001			**44.3** (41.5–NA)/**40.2** (37.9–44.2) HR: 1.0 (0.8–1.23), p = 1	[23]
	2nd line	PAL + FVT vs FVT (after previously having progressed on an AI in metastatic setting)	410/1183		310/310	PSM: 30.1/105.7			**10.0** (8.4–11.8)/**5.0** (4.4–5.9) HR: 0.51 (0.41–0.64), p < 0.0001			**32.3** (28.1–37.4)/**24.6** (21.8–27.7) HR: 0.67 (0.52–0.87), p < 0.0022	

Bold values indicate the values/results presented and discussed in the article.

AI: Aromatase inhibitor; FVT: Fulvestrant; LET: Ietrozole; NE: Not estimable; NR: Not reached PAL: Palbociclib.

**Table 3.** Single arm studies.

Study (year)	Line of treatment	Treatment regimen	Patients (n)	Follow-up (months)	Median PFS (months [95% CI])	Median OS (months [95% CI])	Ref.
Porte *et al.* (2020)	1st line	PAL + AI or FVT	225	20.7	23.0 (20.8–NR)	NR	[24]
	2nd line	PAL + AI or FVT	85	20.7	13.1 (9.0–18.6)	NR	
Law *et al.* (2022)	1st line	PAL + AI	242	22.4	31.7 months (27.9–NE)	NR	[25]

AI: Aromatase inhibitor; FVT: Fulvestrant; LET: letrozole; NA: Not achieved; NR: Not reached; PAL: Palbociclib.

Only efficacy outcomes, in terms of progression-free survival (PFS) and overall survival (OS), are reported in this review. PFS is defined as the time from initiation of the given treatment regimen until the occurrence of disease progression or death. Disease progression was based on records of radiology, pathology and clinical assessment by the treating physician. OS is defined as the time from initiation of treatment until date of death due to any cause.

All the included studies are retrospective observational real-world studies based on patient data from electronic health records retrieved through different health databases. Thus no randomization regarding treatment assignment has been applied [26]. The comparative studies (n = 4) [^20-23^] address this by using statistical methods as inverse probability treatment weighting (sIPTW) and/or propensity score matching (PSM) on their data in order to balance patient characteristics across the two heterogeneous patient groups. As patient characteristics may have influenced treatment choices in clinical practice, adjusting for this potential confounder helps mitigate the risk of confounding in the study results (confounding bias) [26].

The comparative studies are using the following methods to compare the treatment arms in the study: sIPTW, PSM and unadjusted analysis. Given that the majority of the studies use the sIPTW approach as their primary analysis, only results obtained through this method will be reported in the results section of this review. However, one study is using the PSM approach as primary analysis. For this particular study, results derived from the PSM analysis will be reported instead of sIPTW. Data from the different methods are listed in Table 2.

### Identified studies

Rugo *et al.* conducted a study based on patient data from The Flatiron Health Analytic Database. Efficacy of palbociclib + an aromatase inhibitor (AI) versus AI alone as first-line treatment in US clinical practice was analyzed [20]. 2888 postmenopausal women and men were enrolled in the analysis (ten men were included in the PAL + AI arm, and 19 in the AI arm). After sIPTW adjustment, median follow-up time among patients in the PAL + AI arm and AI alone arm was 23.9 months (interquartile range (IQR), 12.8–38.0) and 24.5 months (IQR, 12.0–42.9), respectively. Median PFS after sIPTW was 19.3 months [95% CI: 17.5–20.7] in the PAL + AI group versus 13.9 months [95% CI: 12.5–15.2] in the AI group (HR: 0.70 [0.62–0.78]; p < 0.0001).

The study reported a significantly longer median OS after sIPTW adjustment of 49.1 months [95% CI: 45.2–57.7] in the group of patients treated with PAL + AI versus 43.2 months [95% CI: 37.6–48.0] in the AI-treated group (HR: 0.76 [0.65–0.87]; p < 0.0001) [20].

Another study, based on data from the Flatiron Database, compared efficacy of first-line treatment with palbociclib + letrozole versus letrozole alone among 1430 women treated in a US clinical practice [21]. The primary end point assessed was PFS, and as secondary end point OS. Median follow-up time after sIPTW adjustment was 24.2 months (IQR, 14.2–34.9) in the patient group receiving PAL + LET and 23.3 months (IQR, 12.7–34.3) in the patient group receiving LET alone. After sIPTW, the median PFS of patients treated with PAL + LET was 20.0 months [95% CI: 17.5–21.9], whereas patients treated with LET alone showed a median PFS of 11.9 months [95% CI: 10.5–13.7] (HR: 0.58 [0.49–0.69]; p < 0.0001).

After sIPTW analysis, median OS in the group receiving LET alone was 43.1 months [95% CI: 34.3-NE], while median OS in the PAL + LET group was not reached. (HR: 0.66 [0.53–0.82]; p = 0.0002). The calculated hazard-ratio and p-value stated a significant OS-benefit in patients treated with PAL + LET over LET [21].

Brufsky *et al.* compared the efficacy of palbociclib + Letrozole versus Letrozole alone in first line setting among women with lung and/or liver metastasis treated in US routine clinical practice [22]. The study found 551 women eligible for analysis. 353 of them were diagnosed with lung metastasis, 123 with liver metastasis and 75 with both lung and liver involvement. Median follow-up period was 22.6 months for patients in the PAL + LET group and 22.1 months for patients in the LET alone group. The sIPTW adjusted analysis of patients with lung and/or liver metastasis showed a median PFS of 16.1 months [95% CI: 13.0–20.2] in the PAL + LET group, and 9.6 months [95% CI: 7.2–11.0] in the LET group (HR: 0.56 [0.45–0.69]; p < 0.001), thus reaching statistical significance.

Median OS after sIPTW was not reached in the PAL + LET group, while it was 32.4 months [95% CI: 26.0–48.8] in the LET group (HR: 0.58 [0.43–0.77]; p < 0.001). However, a significant OS-benefit in patients with lung and/or liver metastasis receiving PAL + LET compared with LET alone was obtained [22].

In a study by Ha *et al.*, patient data were collected from the database of a single cancer institution in the USA [23]. Data on the patients receiving endocrine therapy alone (comparison group) were collected between 1997–2020. Thus, some patients in the comparison group were treated even before the FDA granted approval of the palbociclib treatment regimen [10].

The study compared PAL + ET versus ET in two different cohorts: first line and second line treatment. The first line therapy cohort included patients receiving PAL + AI (letrozole, anastrozole, exemestane) (n = 778) or AI alone, while the second line therapy cohort included patients receiving PAL + FVT (n = 410) or FVT (n = 1183) after previously having progressed on an AI prescribed in metastatic setting. 61% of the patients in the fulvestrant control group received fulvestrant at a lower dose (250 mg) since the current dose recommendation on 500 mg wasn't approved until 2010.

The control arm (n = 3635) used for comparison with the PAL + AI arm in the first line analysis included both the control arm from the first-line cohort receiving AI alone (n = 2452), and the control arm from the second line cohort receiving FVT alone after prior progression on an AI (n = 1183). For propensity score matching, patients in the PAL + AI and AI arm were matched 708:708, while patients in the PAL + FVT and FVT arm were matched 310:310.

In the first line cohort, median follow-up (after PSM) was 30 months and 119 months in patients receiving PAL + AI versus AI, respectively. Median follow-up in the second line cohort were 30.1 months in the PAL + FVT group versus 105.7 months in the FVT group.

After PSM, median PFS in the first line cohort was 17.4 months (95% CI: 15.9–20.0) in the PAL + AI arm versus 11.1 months (95% CI: 9.9–13.6) in the AI arm (HR: 0.71 [0.6–0.84]; p < 0.0001). Median OS was 44.3 months (95% CI: 41.5-NA) in the PAL + AI arm versus 40.2 months (95% CI: 37.9–44.2) in the AI arm (HR: 1.0 [0.8–1.23]; p = 1). Thus no significant OS-benefit was found in this cohort. However, the sIPTW analysis showed a HR of 0.79 (0.67–0.93) suggesting that OS was significantly improved in these patients.

After PSM, median PFS in the second line cohort was 10.0 months (95% CI: 8.4–11.8) in the PAL + FVT arm, and 5.0 months (95% CI: 4.4–5.9) in the FVT alone arm (HR: 0.51 [0.41–0.64]; p < 0.0001). Median OS was 32.3 months (95% CI: 28.1–37.4) in the PAL + FVT arm, and 24.6 months (95% CI: 21.8–27.7) in the FVT alone arm (HR: 0.67 [0.52–0.87]; p < 0.0022). Thus, a significantly prolonged median PFS and OS was found in the cohort [23].

Porte *et al.* conducted a single-arm study assessing the effect of palbociclib in combination with endocrine therapy as first or second line treatment among pre- or postmenopausal women [24]. A luteinizing hormone-releasing hormone (LHRH) agonist was prescribed and added to the treatment regimen in the pre-menopausal women (n = 57). The study enrolled 310 patients treated in clinical practice at a single cancer center in France. A total of 225 patients received the palbociclib combination therapy as first line of treatment, while 85 were treated in second line. In 207 patients, the endocrine therapy received was an aromatase inhibitor (letrozole, anastrozole or exemestane), while 103 patients received fulvestrant.

15 patients initiated palbociclib at a lower dose, and ten patients were first prescribed ET alone until palbociclib was also added to the treatment regimen after a median of 51.5 days. Median follow-up was 20.7 months.

The study reported a median PFS of 23.0 months (95% CI: 20.8-NR) in the patients receiving palbociclib in combination with endocrine therapy in first-line setting, while the median PFS in second line setting was 13.1 months (95% CI: 20.8-NR). Median OS was not reached. The study did not distinguish between the received endocrine therapy in the results [24].

Another single-arm study investigated the efficacy of palbociclib in combination with an AI as first-line treatment [25]. Patient data were retrieved from the Syapse Learning Health Network database containing data on patients treated in clinical practises across the US. The study enrolled 242 patients, of which four were men. Women of any menopausal status were included, however the postmenopausal women ended up constituting the largest group of the evaluated population. 25 patients initiated the palbociclib treatment at a lower dose. Median follow-up time was 22.4 months (IQR, 13.1–33.7).

The study found a median PFS of 31.7 months (95% CI: 27.9-NE). In a subanalysis of the patients who presented with advanced or metastatic *de novo* disease, the median PFS was 38.8 (95% CI: 26.5-NE). Patients with recurrent breast cancer didn't gain the same benefit, as their median PFS was 30.5 months (95% CI: 17.4-NE). For patients with bone-only metastasis, median PFS was 44.9 months (95% CI: 39.4-NE). Due to small sample size and limited follow-up time, median OS was not reached [25].

## Discussion

All studies included an evaluation of palbociclib in combination with endocrine therapy in the first line setting. The median PFS in the palbociclib group varied between 16.1–31.7 months, while follow-up ranged from 10.8–30.0 months. Leaving out Law *et al.* (median PFS = 31.7), the range of median PFS among the studies was 16.1–23.0 months. In general, this is shorter than the 27.6 months reported in the extended follow-up analysis of PALOMA-2 [11]. In all studies but one [24], the prescribed endocrine therapy was an AI.

Law *et al.* reported the longest median PFS among the studies – longer than the 27.6 months in PALOMA-2 extended follow-up [11]. These observations are possibly due to the population having a higher proportion of patients with bone-only metastasis (50.8%) and patients presenting with *de novo* metastatic BC (56.6%), compared with patients in the other studies [^20-24^] and PALOMA-2 [8,11].

Brufsky *et al.* evaluating patients exclusively with lung and/or liver metastasis receiving PAL + LET, reported a median PFS of 16.1 months, thus the shortest among the studies. However, this outcome is probably a result of the patients all having visceral metastasis and therefore a poorer prognosis. In addition to this, 4.2% had brain metastasis, which is the highest proportion reported among the studies. The subgroup analysis of patients with visceral metastasis in PALOMA-2 [11] reported a median PFS of 19.3 months in the PAL + LET group, which was considerably shorter than the 27.6 months for the whole population. Thus, results from Brufsky *et al.* are consistent with these subgroup findings in PALOMA-2.

All comparative studies [^20-23^] reported a significantly prolonged median PFS in the PAL + AI/LET group compared with the AI/LET alone group. This is consistent with the findings in PALOMA-2 although the median PFS in the studies were shorter than in PALOMA-2 [11]. One explanation could be the higher age of the patients included in the real-world studies. In the study by Rugo *et al.* 61% of the patient population were aged ≥65, resulting in a median age of 67. The same tendency is seen in DeMichele *et al.* with a median age of 66 years old. In comparison, median age in PALOMA-2 was 62 years old, with only 40.8% of the patients being older than 65 years of age.

The study by Ha *et al.* had the lowest proportion of patients with *de novo* metastatic disease among the real-world studies [^20-22^,^24^,^25^] and PALOMA-2. This could possibly explain the shorter PFS. PSM was used to balance patient characteristics, whereas the other comparative studies were using sIPTW. The study only reported few characteristics of the patients. Consequently, important patient variables potentially causing confounding may not be accounted for in the PSM analysis. Furthermore, the study relied on historical controls in the AI group, which could have affected the results as well.

Porte *et al.* reported one of the higher PFSs. The study however has some limitations. Patients were prescribed either an AI or FVT in combination with palbociclib, but results were not reported for either treatment respectively. Furthermore, characteristics of the patients in the first- and second line cohort respectively were not reported separately, making it difficult to compare the first-line cohort to the one in PALOMA-2.

Only the four comparative studies [^20-23^] were able to report overall survival results in the first-line setting. Two of the studies [21,22] did not reach median OS in the PAL + LET arm, only in the LET alone arm. Still, they managed to report a significant OS benefit in the PAL + LET group compared with the LET group.

Rugo *et al.* [20] showed a significantly prolonged median OS in the PAL + AI group compared with the AI group, while Ha *et al.* [23] failed to show any significant difference after PSM analysis, although still reporting a numerical prolongation of 4.1 months.

Contrary to findings in Rugo *et al.* [20], DeMichele *et al.* [21] and Brufsky *et al.* [22], the survival analysis in PALOMA-2 [14] did not report any significant prolongation of median OS in the patients receiving palbociclib, compared with the control arm.

The two studies reaching median OS in the PAL + AI group reported a median OS of 49.1 months [20] and 44.3 months [23], respectively. These results are noticeably lower than the 53.9 months reported in PALOMA-2. However, it is expected for the highly selected patient population in the clinical trial to live longer compared with the unselected population in the real-world studies.

Additionally, an important point to be considered is that any subsequent therapy received after progression on palbociclib could have affected the OS outcome in both the real-world studies and PALOMA trials.

Only two studies [23,24] evaluated palbociclib in the second line setting. One study [23] evaluated PAL + FVT, while one study [24] evaluated PAL + FVT/AI. Median PFS ranged between 10.0–13.1 months, thus slightly longer than the reported 9.5 months in PALOMA-3 [9].

Porte *et al.* reported a median PFS of 13.1 months. Patients were treated with palbociclib in combination with either FVT or AI but were not distinguished between in the results.

This possibly could have affected the results, as all patients in PALOMA-3 received FVT as the endocrine therapy. Furthermore, 54% of the patients in PALOMA-3 had received two or more previous lines of endocrine therapy, and thus were more heavily pretreated.

The second-line cohort in Ha *et al.* reported a median PFS of 10 months in the patients receiving PAL + FVT compared with 5 months in the FVT group, reaching statistical significance. Thus, results from this study were consistent with the findings in PALOMA-3. The patients had progressed on AI in metastatic setting, thus this real-world cohort was similar to the one in PALOMA-3.

Ha *et al.* was the only study reporting median OS in patients treated in second line with palbociclib. A median OS of 32.3 months was found in the PAL + FVT group, which is in close proximity to findings in PALOMA-3 where a median OS of 34.8 months was reported.

In the real-world study a significant increase in median OS was reported favoring PAL + FVT compared with FVT. In PALOMA-3 however, no significant OS benefit was found [12,15].

The studies included in this review have several limitations relating to the retrospective design including the lack of randomization [26]. The comparative studies [^20-23^] addressed this by using sIPTW and/or PSM in order to balance patient characteristics (e.g., age, site of metastasis etc.) across the two groups examined in the study. However, only the patient characteristics identified as possible confounders are accounted for in the adjusted analysis. Thus, if any unidentified patient variables, potentially able to affect treatment assignment, are present, these will not be accounted for. As a result, these might affect and confound the findings in the studies. In RCTs, the randomization automatically corrects for both observed and unobserved potential confounders [26].

The studies were based on data from electronic health records retrieved from different databases. Data were collected in routine clinical practice, thus, without a research intention and outside the controlled conditions of the RCTs. Patient records could possibly contain errors in the data entered by the physician, and some data might never have been collected at all. In addition, disease progression was based entirely on the treating physician's individual assessment of radiology and pathology results.

Two studies had no comparison group as they were single arm studies, thus limiting interpretation of the study results [24,25].

Only few studies reached median OS in the palbociclib group, suggesting the need of longer follow-up and larger population sizes in the real-world studies [20,23].

All studies except one [24] based their analysis on patients treated in clinical practices in the USA. This possibly limits the applicability of the results to patient populations outside the USA. Two studies [23,24] based their analysis on patient data from a single cancer center. In addition, the database used for retrieval of patient records also differed between the studies in this review. Thus, all these factors potentially limit the application of the study-findings to patient populations outside the one investigated in each study.

The randomized controlled trials investigating palbociclib, ribociclib and abemaciclib, which led to FDA approval, consistently demonstrated a statistically significant improvement in PFS irrespective of treatment line. However, as OS results have matured and been reported, noticeable differences among the trials have appeared.

The PALOMA trials failed to report any significant OS benefit from palbociclib [12,14]. Meanwhile, the MONALEESA-3 trial investigating ribociclib reported a significant OS benefit, while also reporting the longest median OS among the pivotal trials (67.6 months) [27]. Data from the second interim analysis in MONARCH-3 investigating abemaciclib in the first-line setting have shown a numerical benefit in OS without statistical significance, but final results are pending [28]. Thus, findings in the trials suggest potential variations among the different CDK4/6i's, pointing toward a shift in selection preferences [29].

In some countries, the lack of significant OS benefit from palbociclib has led to a change in national guidelines favoring ribociclib and abemaciclib over palbociclib [30].

Comparative real-world studies [^20-22^] report a significant improvement in OS. Thus, results from the real-world studies on palbociclib seem to contradict findings from the pivotal trials. As further real-world studies on palbociclib with longer follow-up are conducted, it will allow for additional insights on the real-world efficacy of palbociclib. However, as of now, real-world studies support the use of palbociclib as an effective treatment option for patients in real-world clinical practice.

The three CDK4/6i's differ in terms of their toxicity profile. Ribociclib exhibits a higher risk of liver toxicity and QTc prolongation compared with abemaciclib and palbociclib. Meanwhile, abemaciclib is associated with a higher risk of gastrointestinal toxicity than the other two CDK4/6i's [31]. As some patients might not tolerate ribociclib or abemaciclib, it is desirable from a clinical point of view, that the best tolerated CDK4/6i is not deemed ineffective unless strong data support such a conclusion.

It is crucial, that larger real-world studies on ribociclib and abemaciclib are conducted and reported in the future, to ensure available real-world data on all three CDK4/6i's. In addition, both real-world studies and pivotal trials comparing the CDK4/6i's head-to-head are needed. An ongoing trial, HARMONIA (NCT05207709), will be the first study to randomize patients with mBC to receive either palbociclib or ribociclib. However, this trial focuses on patients with the HER2-enriched subtype. In a recent study directly comparing the three CDK4/6i's in terms of PFS and OS in a real-world population, no statistically significant differences were observed among the agents [32].

Given the absence of real-world studies regarding the effectiveness of ribociclib and abemaciclib, the current landscape of CDK4/6i data should be interpreted with caution while awaiting further real-world data on ribociclib and abemaciclib.

## Conclusion

In general, real-world studies indicate that palbociclib in combination with endocrine therapy is associated with improved PFS and OS outcomes in patients with HR+/HER2- mBC in both first- and second-line setting.

Three comparative studies found a statistically significant improvement in OS when palbociclib was given in the first-line setting in combination with an AI. As data from the PALOMA-2 trial failed to report any statistically significant OS-benefit, real-world studies seem to contradict the results from phase III trials with palbociclib.

Thus, real-world studies support palbociclib as an effective treatment option in real-world clinical practice. However, additional real-world studies on palbociclib with longer follow-up are still needed.

It is clear that patient populations in real-world studies exhibit significant heterogeneity and often differ from those in clinical trials. As a consequence, comparisons among real-world studies and between real-world studies and RCTs pose certain challenges.

## Future perspective

As palbociclib was the initial CDK4/6i to receive FDA approval, the majority of the current available real-world data revolves around this specific CDK4/6i. However, as real-world experience with ribociclib and abemaciclib increases, real-world efficacy data are starting to emerge. A recent study failed to show any significant difference in OS among the three CDK4/6i's. Given the lack of clinical understanding as to why the CDK4/6i's should perform differently in similar populations, real-world data on ribociclib and abemaciclib will be highly anticipated. Hopefully the near future will provide crucial insight on how these CDK4/6i's compare in real-world populations, and whether these results align with findings from the pivotal trials.

Facing the still unresolved question whether a given OS benefit is attributable to CDK4/6 inhibitors as a class of drugs or is associated with specific drugs, it is paramount that both real-world studies and randomized trials directly comparing the three CDK4/6i's are conducted and reported in the future.
